# Isolation and Sequencing of Chromosome Arm 7RS of Rye, *Secale cereale*

**DOI:** 10.3390/ijms231911106

**Published:** 2022-09-21

**Authors:** Jakob Petereit, Cassandria Tay Fernandez, Jacob I. Marsh, Philipp E. Bayer, William J. W. Thomas, Aybeniz Javad Aliyeva, Miroslava Karafiátová, Jaroslav Doležel, Jacqueline Batley, David Edwards

**Affiliations:** 1 School of Biological Sciences, The University of Western Australia, Perth 6009, Australia; 2 Molecular Cytogenetics Department, Genetic Resources Institute of Azerbaijan National Academy of Sciences, Baku 1106, Azerbaijan; 3 Institute of Experimental Botany of the Czech Academy of Sciences, Centre of the Region Haná for Biotechnological and Agricultural Research, Šlechtitelů 31, CZ-77900 Olomouc, Czech Republic

**Keywords:** rye, isolated chromosome arm sequencing, presence-absence variation

## Abstract

Rye (*Secale cereale*) is a climate-resilient cereal grown extensively as grain or forage crop in Northern and Eastern Europe. In addition to being an important crop, it has been used to improve wheat through introgression of genomic regions for improved yield and disease resistance. Understanding the genomic diversity of rye will assist both the improvement of this crop and facilitate the introgression of more valuable traits into wheat. Here, we isolated and sequenced the short arm of rye chromosome 7 (7RS) from Triticale 380SD using flow cytometry and compared it to the public Lo7 rye whole genome reference assembly. We identify 2747 Lo7 genes present on the isolated chromosome arm and two clusters containing seven and sixty-five genes that are present on Triticale 380SD 7RS, but absent from Lo7 7RS. We identified 29 genes that are not assigned to chromosomal locations in the Lo7 assembly but are present on Triticale 380SD 7RS, suggesting a chromosome arm location for these genes. Our study supports the Lo7 reference assembly and provides a repertoire of genes on Triticale 7RS.

## 1. Introduction

Rye (*Secale cereale* L., 2*n* = 2*x* = 14, RR genome) is a valuable cereal grain and commercial crop, processed for culinary uses in bread, beer and whisky, consumed raw as animal fodder and has utility in the field as a cover crop [1]. It is a stress tolerant cereal known for its resilience in low fertility soils [2,3]. As a member of the Triticeae tribe in the grass family Poaceae, it is closely related to modern domesticated wheat [4], having diverged from a common ancestor only 7 million years ago [5,6]. Similar to *Aegilops* spp. [7] and *Agropyron* spp. [8], rye chromatin can be recombined with wheat chromatin, allowing the translocation of DNA segments between the two species. The introduction of rye DNA into wheat has been exploited to improve wheat varieties [9]. For example, recombination between wheat 1AL and rye 1RS resulted in greenbug resistance, and recombination between wheat 1BL and rye 1RS improved yield as well as stem stripe and powdery mildew resistance [10,11]. The ability to transfer beneficial traits to wheat varieties increases the agronomic value that can be achieved through the identification and characterization of the rye genome. Thoroughly characterizing the rye genome is therefore important, not only for rye improvement, but also for the introduction of beneficial rye traits into wheat.

Rye has the largest genome of all temperate cereals, with a size of approximately 7.9 Gb/1C [12,13]. The amplification of transposable elements was a major cause of this expansion, and these represent over 90% of the genome [14,15]. Rye’s large genome size and repetitive content impeded the development of a genome assembly, even after the advent of NGS technology. However, two long awaited high-quality chromosome scale reference assemblies were published in 2021, the rye inbred line ‘Lo7’ [16] and Weining rye [17], with assembly sizes of 6.74 Gb and 7.74 Gb, respectively. Lo7 is a German winter rye, which is adapted to the major global rye producing regions in north-eastern Europe [18]. Weining rye is an early flowering variety cultivated in China which has broad-spectrum resistance to common wheat diseases, including powdery mildew and stripe rust [19,20]. The availability of these assemblies will support investigation into rye’s domestication history, aid in identifying the genomic basis of agronomically important traits and assist in the transfer of these traits into wheat.

Once a genome has been assembled, the assembly can be assessed and validated through the physical isolation and sequencing of chromosome arms, and mapping of the resulting reads to the assembly [21]. Whole chromosome and chromosome arm sequencing has supported the assembly of the genomes of wheat [22,23,24] and pea [25]. Chromosome arm sequencing validates the genomic positions and context of QTLs located on chromosome arms [26]. Rye chromosome 7 contains QTLs for pre- harvest sprouting [27], grain α-amylase activity [28], flowering time [29], kernel weight and gibberellic and abscisic acid responses [30]. Assessing and validating the genomic sequence of chromosome 7 is therefore valuable, potentially supporting the improve of both rye and wheat [31].

Crossbreeding wheat and rye gave rise to triticale, a wheat-rye hybrid crop that is highly resistant to pathogens including leaf and stem rusts [32], displays high yield and is used as animal fodder and for genomics research. The most common chromosomal alterations found in wheat-rye substitution are usually deletions and translocation of individual chromosomal regions such as chromosome arms [33,34]. Here, we isolate, sequence and characterize a rye chromosome arm present in Triticale 380SD and assess its gene content. The Triticale 380SD genome shows two R/D chromosome substitutions (4R(4D), 5R(5D)) and a ditelosomic addition (7RS). The triticale used as a source for the telocentric chromosome, Triticale ABR, originated from a stabilised cross of synthetic *wheat Triticum durum/Aegilops tauschii* var. *meyeri* with wild rye (*Secale cereale* subsp*. Segetale*), and has been highly modified by different steps of introgression and crossings. The stable crossing of wild rye with synthetic wheat suggests that the centromere and the long arm sequence, 7R, is similar to wild rye, which is its primary source.

Cultivated rye has been incompletely genetically isolated from its wild relatives and rye is known to show large amounts of heterozygosity within and between genomes [16]. However, cereal rye is hypothesised to be a mosaic of different rye species and is not reproductively isolated, being open to hybrid breeding [17]. Many chromosomes are conserved between domesticated rye and wild rye, such as the B chromosomes, which have been well studied and documented [35,36]. Additionally, structural heterozygosity for reciprocal translocations has been reported both in cultivated and wild rye [37]. By comparing chromosome arm 7RS from the rye donor line with the Lo7 chromosome 7 assembly, we identify gene clusters that are lost or moved to new locations after the two lines diverged. Assessing variation on this chromosome will facilitate rye breeding and the introgression of rye traits into wheat.

## 2. Results and Discussion

**Figure 1:**
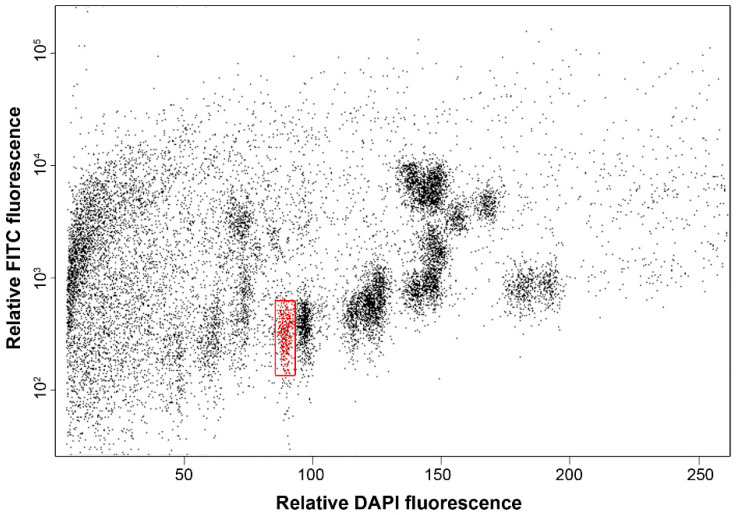
Bivariate flow karyotype FITC vs. DAPI fluorescence obtained after the analysis of chromosomes isolated from the Triticale 380SD line. The population representing chromosome arm 7RS was clearly discriminated and this allowed its sorting using window shown as red rectangle.

Bivariate flow cytometric analysis of chromosomes isolated from the Triticale 380SD line yielded flow karyotype which groups of chromosomes with similar DNA and GAA content formed separated populations (Figure 1). As the 7RS telosome is smaller than the remaining chromosomes of Triticale 380SD line, its population was clearly discriminated. This permitted its flow sorting at average purity of 87%. In order to reduce DNA amplification bias, DNA amplified from both flow-sorted batches was pooled to give 8.37 µg DNA.

**Figure 2:**
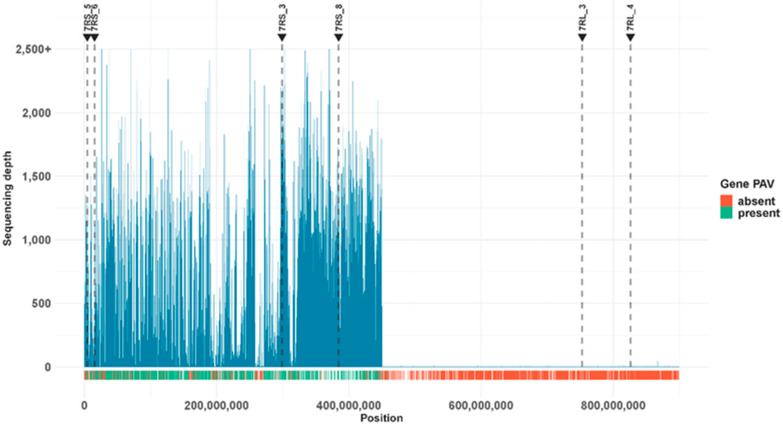
Chromosome arm 7RS Illumina DNA sequence reads were aligned to the Lo7 reference assembly. The median sequencing depth of 100 Kb windows is depicted in blue. Dashed lines indicate the chromosome arm PLUG marker alignment positions. Gene PAV is indicated as red (absent) and green (present) segments.

DNA amplified from the flow-sorted 7RS arm was sequenced generating a total of 142.8 Gb of data. Reads were aligned to the Lo7 reference assembly [16] and 449 Mb, approximately 50% of chromosome 7R, showed high sequence coverage, starting around the third gene on the chromosome, SECCE7Rv1G0453870.1, and terminating around gene SECCE7Rv1G0489010.1 at position 449,957,624 (Figure 2). To validate the sequenced chromosome arm, rye genetic markers [38] were aligned to the Lo7 reference genome assembly. Four 7RS specific markers were located within the high coverage region of chromosome 7 and two 7RL markers in the low coverage region of chromosome 7, confirming the sequenced arm as the short arm of rye chromosome 7 (Figure 2).

**Figure 3:**
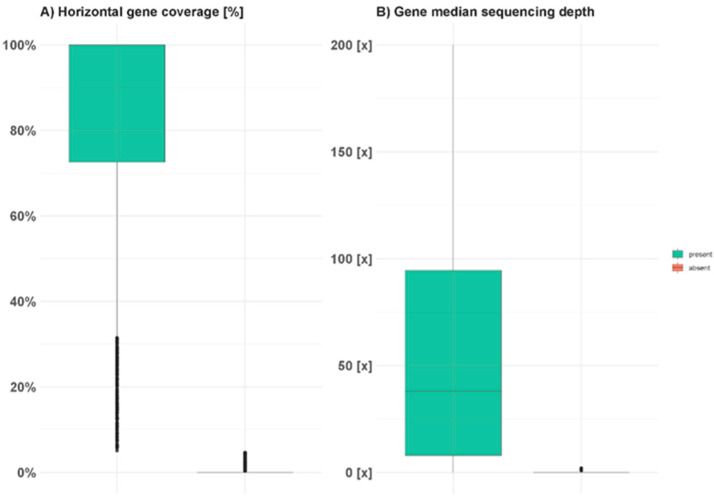
Statistics of rye 7RS genes present and absent on Triticale 380SD 7RS. (**A**) Horizontal gene coverage of bases within the coding gene region of genes on the short arm of rye chromosome 7. (**B**) Median sequencing depth of the coding regions of genes on the short arm of rye chromosome 7.

To evaluate gene content on the isolated chromosome arm, the 449 Mb high coverage region was intersected with the Lo7 reference gene model annotation, resulting in 3517 total Lo7 gene models in the high coverage region. Gene presence was called using a modified version of the SGSGeneLoss pipeline [39] (Figure S1). Of 3,517 genes on Lo7 7RS, 2747 were called as present (Table S1) and showed a median horizontal coverage of 99.9% with a median sequencing depth of 38X, while absent genes showed had a median horizontal coverage of 0% and a median sequencing depth of 0X (Figure 3).

Genes that are present in Lo7 7RS but absent in the sequenced arms formed two clusters from position 197,001,808 to 198,126,643 and from position 258,201,402 to 273,997,788 (Figure 2). In the region from position 197,001,808 to 198,126,643 on 7R, a block of seven consecutive genes were called as absent (SECCE7Rv1G0476080.1, SECCE7Rv1G0476090.1, SECCE7Rv1G0476100.1, SECCE7Rv1G0476110.1, SECCE7Rv1G0476120.1, SECCE7Rv1G0476130.1 and SECCE7Rv1G0476140.1) and all seven genes had zero bases exceeding the 2X base coverage threshold (Figure S2). The Lo7 annotation classifies gene models as high- and low-quality predictions [16], and in this region only two of the seven missing genes had high quality annotation, suggesting that the missing genes may be due to mis-annotation of the Lo7 reference. Of the seven missing genes in cluster one, only two yielded conserved domains, when subjected to the NCBI conserved domain search (Table S3A). The Lo7 7RS region between position 258,401,889 and position 271,512,497 contains 81 genes (from SECCE7Rv1G0479760.1 to SECCE7Rv1G0480790.1), 65 of which are absent from the rye donor chromosome arm. The absence of the 65 genes was confirmed by their per-base coverage, which never exceeded a 2X threshold for 5% of bases in a gene (Figure S3). Of the 65 absent genes, 29 had high quality Lo7 annotations and only four lacked a conserved domain, suggesting that this block of genes may be lost or moved after the divergence of the Lo7 and the rye donor line. The 65 missing genes were subjected to a NCBI conserved domain search and conserved domains included various domains (Table S3B).

The chromosome arm sequencing data can be used to support the allocation of unplaced genes onto chromosome arms. We identified 29 genes that are in annotated in unplaced contigs in the Lo7 reference assembly and had no pseudomolecule assignment but present in our isolated 7RS dataset (Table S2). The presence of these 29 genes was confirmed by their individual per-base coverage (Figure S4). The 29 present genes could be added to Lo7 7RS in future versions of reference quality rye assemblies.

## 3. Materials and Methods

### 3.1. Purification of Chromosome Arm 7RS

This study used Triticale 380SD, a line developed by the Genetic Resources Institute of Azerbaijan National Academy of Sciences (ANAS) which was generated by crossing triticale (2*n* = 6*x* = 42, genome AABBRR) and the Chinese Spring wheat variety in 1990, and the results of the molecular cytogenetic study were published in 2020. Triticale 380SD’s maternal parent is Triticale ABR (also indicated as NA-75) which originated from the stabilised cross of synthetic wheat *Triticum durum*/*Aegilops tauschii* var. *meyeri* (Kyoto, GenBank accession number AD 221-16a by 1975) with *Secale cereale* subsp. *segetale*. The Triticale 380SD genome shows two R/D chromosome substitutions (4R(4D), 5R(5D)) and a ditelosomic addition (7RS). Rye chromosome arm 7RS was purified by flow cytometric sorting from Triticale 380SD as describe in Vrána, Kubaláková [40] and Kubaláková, Vrána [41]. Briefly, root tip cells of young seedlings were synchronized using hydroxyurea, accumulated in metaphase using amiprohos-methyl and fixed by formaldehyde. Intact chromosomes were released by mechanical homogenization of root tips in LB01 buffer [42]. GAA microsatellite clusters on chromosomes in suspension were labelled by FITC using fluorescence in situ hybridization in suspension (FISHIS) as described by Giorgi et al. [43] and chromosomal DNA was stained by DAPI. Chromosome analysis and sorting was conducted using FACSAria II SORP flow cytometer and sorter (Becton Dickinson Immunocytometry Systems, San José, CA, USA). Sort window was setup on a dot-plot FITC vs. DAPI fluorescence (Figure 1) and two batches of 25,000 7RS telosomes were sorted into 40 μL sterile deionized water in PCR tubes. To determine chromosome content of the sorted fractions, 1000 chromosomes were flow sorted into 10 μL of PRINS buffer containing 2.5% sucrose [44] on a microscopic slide, labelled by FISH with a probe for GAA microsatellite and evaluated microscopically.

### 3.2. Sequencing Flow-Sorted 7RS

DNA of flow-sorted chromosome arms 7RS was amplified following Šimková et al. [45]. The chromosomes were treated with proteinase K and their DNA was amplified by multiple displacement amplification using an Illumina GenomiPhi V2 DNA Amplification Kit (GE Healthcare, Chalfont St. Giles, UK). DNA amplified from both batches of flow-sorted 7RS was pooled. The libraries for genome sequencing were prepared using the Illumina Tru-seq Nano DNA HT Library Preparation kit, according to the manufacturer’s instructions. Genomic DNA was sequenced using an Illumina XTEN sequencer with 150 bp paired-end (PE) technology at the Garvan Institute of Medical Research. Sequence data was cleaned and trimmed using Trimmomatic-0.36 [46] to remove low quality regions and adaptors.

### 3.3. Presence-Absence Variation Analysis

The rye Lo7 reference genome assembly was downloaded from (https://doi.org/10.5447/ipk/2020/33, accessed 18 February 2018) [16]. Bowtie2 v2.4.2 was used to align the sequence reads [47]. Sam files were filtered for a minimum quality score of 30. The sam files were then converted to bam files, sorted and their per-base coverage was calculated using SAMtools v1.11 [48]. Rye specific PLUG markers were downloaded (https://doi.org/10.1007/s00412-013-0428-7, accessed 18 February 2018) and aligned to the Lo7 reference genome using Bowtie2 [38].

Presence-absence variation analysis was performed using the SGSGeneLoss pipeline [39], in short, the prior calculated per-base sequencing depth for each gene was filtered for a minimum of 2X and used to calculate the relative amount of coding sequence bases which exceed the cut off for each gene (horizontal coverage). Genes exhibiting more than 5% horizontal coding classified as present. To identify genes that were placed on unplaced contigs in the Lo7 reference assembly, a more conservative SGSGeneLoss cutoff of 50X minimum per-base sequencing depth, 95% horizontal coverage was applied.

### 3.4. Data Analysis

Data analysis was performed using R v4.1.0 [49], Rstudio v 1.4.1717 [50] and the R packages ‘Tidyverse’ v1.3.1 [51] and ‘data_table’ v1.14.0 [52]. Data visualisation was performed using the ggplot function in Tidyverse. Median coverage for 100 Kb windows was calculated and visualized using a custom Rscript.

## 4. Conclusions

The Lo7 reference genome is a profound foundation to identify candidates and isolated chromosome sequencing can validate physical gene positions and provide the genetic material for introgression at the same time. Missing genes identified in this study were likely moved or removed after divergence of Triticale 380SD and should be carefully validated within any introgression donor line, specifically, the rye donor used in the generation of Triticale ABR (NA-75). Once validated if the missing genes are mis-annotations or not, these segments can be used as markers for future introgression studies. Finally, identifying the 29 genes present in the isolated 7RS dataset which are located in Lo7 unplaced contigs will further enable wheat introgression.

## Data Availability

Raw reads are available at the Sequence Read Archive (SRA) under BioProject PRJNA835423.

## Supplementary Materials

The following supporting information can be downloaded at: https://www.mdpi.com/article/10.3390/ijms231911106/s1.
