# Supplementary material for: Isolation and Sequencing of Chromosome Arm 7RS of Rye, Secale cereale
Source: Int J Mol Sci. 2022 Sep 21;23(19):11106. doi: 10.3390/ijms231911106 (PMC9569962; doi:10.3390/ijms231911106)
Supplement: Supplementary file 1 [file ijms-23-11106-s001.zip › Figure S3 - cluster2 missing genes.pdf]

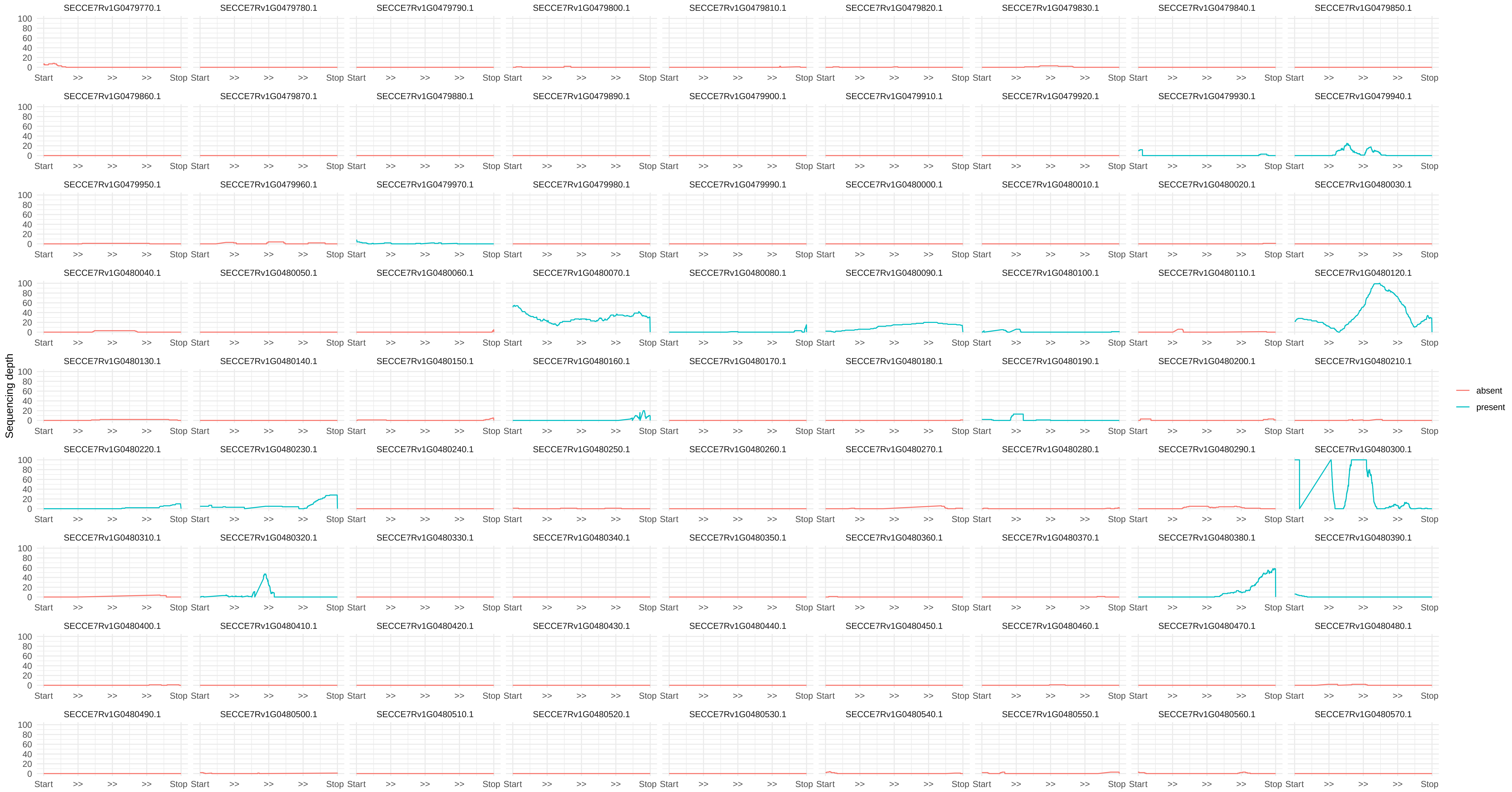

Figure S3 − Cluster 2 gene sequencing depth. X axis depicts
gene CDS bases, y axis the per−base sequencing depth, capped at 100X.
