# Supplementary material for: Isolation and Sequencing of Chromosome Arm 7RS of Rye, Secale cereale
Source: Int J Mol Sci. 2022 Sep 21;23(19):11106. doi: 10.3390/ijms231911106 (PMC9569962; doi:10.3390/ijms231911106)
Supplement: Supplementary file 1 [file ijms-23-11106-s001.zip › Figure S4 - unplaced lo7 genes present on isolated 7RS.pdf]

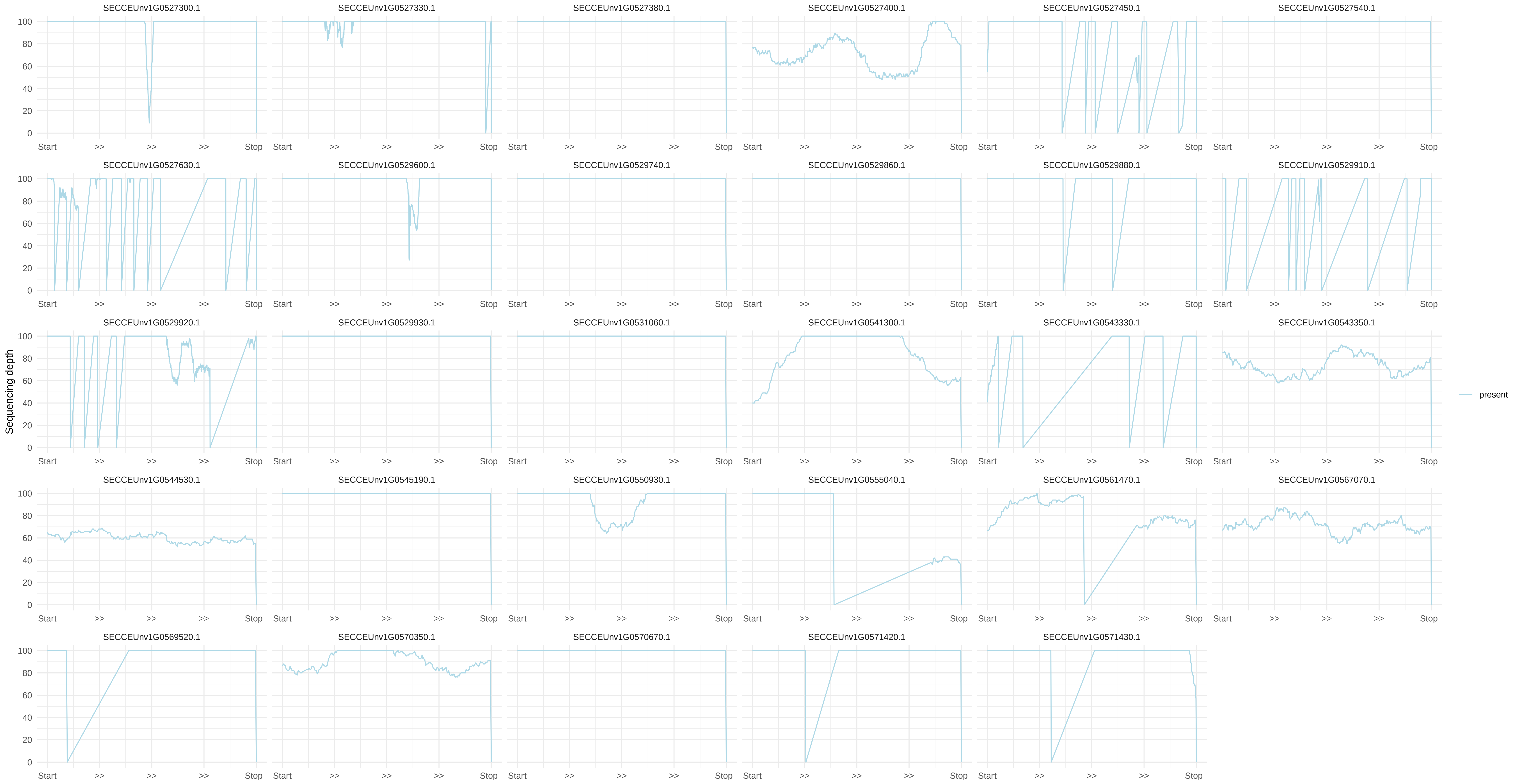

Figure S4 − lo7 genes that are located in unlaced contigs in the lo7 refrence assembly, but were called present on the on the isolated chromsome arm 7RS. X axis depicts gene CDS bases, y axis the per−base sequencing depth, capped at 100X.
