# Supplementary figures and images for: Isolation and Sequencing of Chromosome Arm 7RS of Rye, Secale cereale
Source: Int J Mol Sci. 2022 Sep 21;23(19):11106. doi: 10.3390/ijms231911106 (PMC9569962; doi:10.3390/ijms231911106)

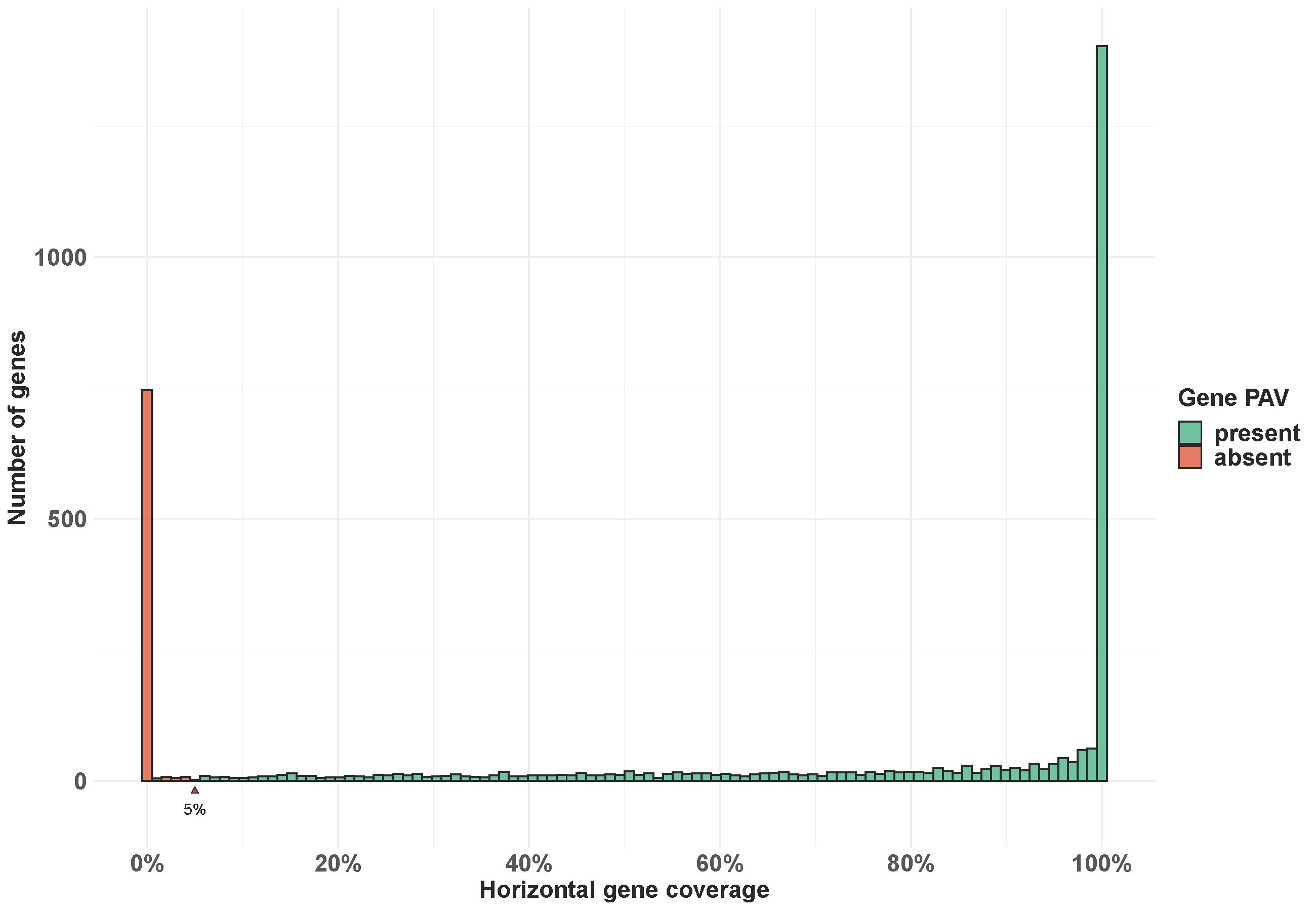

Supplement: Supplementary file 1 [file ijms-23-11106-s001.zip › Figure S1 - PAV_7RS_histogram.jpg]
